# ASK1 is a novel molecular target for preventing aminoglycoside-induced hair cell death

**DOI:** 10.1007/s00109-022-02188-1

**Published:** 2022-04-26

**Authors:** Jacqueline M. Ogier, Yujing Gao, Eileen M. Dunne, Michael A. Wilson, Sarath C. Ranganathan, Gregory H. Tesch, David J. Nikolic Paterson, Alain Dabdoub, Rachel A. Burt, Bryony A. Nayagam, Paul J. Lockhart

**Affiliations:** 1 Bruce Lefroy Centre, Murdoch Children’s Research Institute, Parkville, VIC Australia; 2 Department of Paediatrics, University of Melbourne, Parkville, VIC Australia; 3 Sunnybrook Research Institute, Toronto, ON Canada; 4 Pneumococcal Research Group, Murdoch Children’s Research Institute, Parkville, VIC Australia; 5 Infection and Immunity, Murdoch Children’s Research Institute, Parkville, VIC Australia; 6 Department of Respiratory Medicine, The Royal Children’s Hospital, Parkville, VIC Australia; 7 Department of Nephrology, Monash Health and Monash University Centre for Inflammatory Diseases, Clayton, VIC Australia; 8 Department of Anatomy and Neuroscience, The University of Melbourne, Parkville, Australia; 9 Department of Audiology and Speech Pathology, The University of Melbourne, Parkville, VIC Australia

**Keywords:** Hair cell, Ototoxic, MAP3K5, p38, JNK, Hearing

## Abstract

**Abstract:**

Aminoglycoside antibiotics are lifesaving medicines, crucial for the treatment of chronic or drug resistant infections. However, aminoglycosides are toxic to the sensory hair cells in the inner ear. As a result, aminoglycoside-treated individuals can develop permanent hearing loss and vestibular impairment. There is considerable evidence that reactive oxygen species (ROS) production and the subsequent phosphorylation of c-Jun N-terminal kinase (JNK) and P38 mitogen-activated protein kinase (P38) drives apoptosis in aminoglycoside-treated hair cells. However, treatment strategies that directly inhibit ROS, JNK, or P38 are limited by the importance of these molecules for normal cellular function. Alternatively, the upstream regulator apoptosis signal-regulating kinase 1 (ASK1/MAP3K5) is a key mediator of ROS-induced JNK and P38 activation under pathologic but not homeostatic conditions. We investigated ASK1 as a mediator of drug-induced hair cell death using cochlear explants from *Ask1* knockout mice, demonstrating that *Ask1* deficiency attenuates neomycin-induced hair cell death. We then evaluated pharmacological inhibition of ASK1 with GS-444217 as a potential otoprotective therapy. GS-444217 significantly attenuated hair cell death in neomycin-treated explants but did not impact aminoglycoside efficacy against *P. aeruginosa* in the broth dilution test. Overall, we provide significant pre-clinical evidence that ASK1 inhibition represents a novel strategy for preventing aminoglycoside ototoxicity.

**Key messages:**

- ASK1 is an upstream, redox-sensitive regulator of P38 and JNK, which are known mediators of hair cell death.
- *Ask1* knockout does not affect hair cell development in vivo, but significantly reduces aminoglycoside-induced hair cell death in vitro.
- A small-molecule inhibitor of ASK1 attenuates neomycin-induced hair cell death, and does not impact antibiotic efficacy in vitro.
- ASK1 may be a novel molecular target for preventing aminoglycoside-induced hearing loss.

**Supplementary information:**

The online version contains supplementary material available at 10.1007/s00109-022-02188-1.

## Introduction

Aminoglycoside antibiotics are used for serious or drug resistant infections of the respiratory tract, urinary tract, central nervous system or intra-abdominal organs, as well as endocarditis or sepsis (in adults and neonates), and prophylactically during and post-surgery. Aminoglycoside therapies preserve life; however, negative side effects are associated with their use. Careful dosing and monitoring regimens limit toxic outcomes, including encephalopathy, neuromuscular blockade, and nephrotoxicity [1]. However, ototoxicity (toxic outcomes relating to the ear) remains a persistent problem.

Aminoglycoside ototoxicity predominantly manifests as irreversible damage to the sensory hair cells (HCs) of the cochlea and vestibular apparatus. Therefore, aminoglycoside-treated individuals can experience permanent damage to their hearing and/or balance. The reported prevalence of aminoglycoside-induced ototoxic outcomes is variable (Table 1). Nevertheless, for those affected, ototoxicity has a profound effect on their quality of life [2, 3]. Currently, no treatment exists for the prevention of aminoglycoside-induced ototoxicity. Instead, clinical practice aims to minimise or avoid aminoglycoside use. However, an alternate therapy is not always feasible and a single aminoglycoside treatment can cause permanent damage to the inner ear [4]. Therefore, research efforts have focused on elucidating the mechanisms underlying aminoglycoside-induced HC death, to identify therapeutic targets to mitigate ototoxicity. These studies have established that HCs are susceptible to aminoglycoside toxicity, due to the abundance of HC specific mechano-electro-transduction (MET) channels that facilitate rapid aminoglycoside entry into the cell [5, 6]. Once the aminoglycoside has entered the HC, it targets and damages the mitochondrial ribosome, preventing mitochondrial protein synthesis.

**Table 1:** The reported prevalence of ototoxic outcomes in humans treated with aminoglycosides. The American Speech Language Hearing Association (ASHA) definition of ototoxicity is described as a hearing threshold shift ≥ 20 dB in one frequency; ≥ 10 dB at any two adjacent frequencies, or loss of response at three consecutive frequencies [25]. However, numerous studies use different definitions of ototoxicity

Aminoglycoside	Prevalence of ototoxic outcomes	Number in sample	Described method for identifying ototoxicity	Definition of ototoxicity	Average duration of therapy	Dosage	Ref
Amikacin	75%	38/51	Audiometry 250–8000 Hz	Hearing was classified as normal (0–20 dB) or impaired (≥ 21 dB) using the Namibian Ministry of Health & Social Services audiogram chart	6–8 months	15 mg/kg/day	[26]
Amikacin	70%	29/41	Audiometry 250–8000 Hz	ASHA 1994 Definition	45 days (range 13–131)	500 mg/day	[27]
Amikacin	62%	270/437	High frequency audiometry or diagnosed by the treating physician	Hearing threshold shift ≥ 15 dB at two or more frequencies or an increase ≥ than 20 dB at one frequency; or the absence of usable hearing acquired during or soon after amikacin treatment	167 days (range 105–210)	15 or 25 mg/kg/day	[28]
Amikacin	55%	12/22	Audiometry 250–8000 Hz	Neurosensory hearing loss ≥ 20 dB at any frequency	20 weeks (range 1–43)	75 mg/kg/week	[29]
Amikacin	51%	20/39	Audiometry 250–8000 Hz	Hearing threshold shift ≥ 15 dB at one frequency in one ear or ≥ 10 dB at three frequencies in one ear	20 days (range 4–83)	7.5 mg/kg/12 h	[30]
Amikacin	33%	13/39	Audiometry 250 Hz–20 kHz	ASHA 1994 definition	> 4 days	not defined	[31]
Amikacin	30%	7/23	Audiometry 250–8000 Hz, caloric‐evoked nystagmus & cVEMP	Not defined	40 days (range 15–84)	7.5 mg/kg/8 h	[32]
Amikacin	35%	6/17	Audiometry 250 Hz–20 kHz	Hearing threshold shift ≥ 15 dB at two frequencies	8 days ± 3.3	7.5 mg/kg/12 h	[33]
Amikacin	25%	6/24	Audiogram	ASHA 1994 definition	28 days	15 mg/kg/day	[34]
Amikacin	21%	6/28	Caloric stimulation	No response to caloric test	3 days	15 mg/kg/day	[35]
Amikacin	18%	7/40	Audiometry 250–8000 Hz	Hearing thresholds outside the normal 0–15 dB range for children at 6–8 kHz	5 days ± 2	15 mg/kg/day	[36]
Amikacin	9%	4/44	Audiometry 250–8000 Hz	Hearing threshold shift ≥ 20 dB at any frequency	138 days (IQR 70–187)	400 mg/day	[37]
Amikacin	7%	4/60	Audiometry 250–8000 Hz	Hearing threshold shift ≥ 15 dB at two or more frequencies	10 days	15 mg/kg/day	[38]
Gentamicin	53%	8/15	Prodromal questioning for vestibular signs & high frequency audiometry	Hearing threshold shift ≥ 15 dB at two frequencies in the same ear or ≥ 10 dB in two frequencies in both ears. Patients also asked about other ototoxic signs including tinnitus, dizziness, or bobbing oscillopsia	8 days ± 4	4 mg/kg/day	[39]
Gentamycin	34%	116/339	Audiometry 250 Hz–20 kHz	ASHA 1994 definition	> 4 days	Not defined	[31]
Gentamicin	13%	14/106	Audiometry 250–8000 Hz	Hearing threshold shift ≥ 15 dB at both 6 & 8 kHz	5–7 days	80–160 mg/12 h	[40]
Kanamycin	56%	168/302	Audiometry 250–8000 Hz	Hearing was classified using the Namibian Ministry of Health & Social Services audiogram chart. Normal = 0–20 dB. Impaired ≥ 21 dB	6–8 months	15 mg/kg/day	[26]
Kanamycin	42%	14/33	Audiometry 250–8000 Hz	Neurosensory hearing loss ≥ 20 dB at any frequency	14 weeks (range 1–139)	75 mg/kg/week	[29]
Kanamycin	22%	5/23	Audiometry 250–8000 Hz	Hearing threshold shift ≥ 20 dB at any frequency	104 days (range 82–180)	400 mg/day	[37]
Kanamycin	20%	9/42	Audiometry 250–8000 Hz	Hearing threshold shift ≥ 20 dB in one frequency or ≥ 15 dB at any two adjacent frequencies	10 weeks ± 5	Not defined	[41]
Netilmicin	47%	7/15	Prodromal questioning for vestibular signs & high frequency audiometry	Hearing threshold shift ≥ 15 dB at two frequencies in the same ear or ≥ 10 dB in two frequencies in both ears. Patients also asked about other ototoxic signs including tinnitus, dizziness, or bobbing oscillopsia	11 days ± 4	5.5 mg/kg/d	[39]
Netilmicin	4%	3/68	Audiometry 250–8000 Hz	Hearing threshold shift ≥ 15 dB at two frequencies	8 days ± 3	1.7 mg/kg/ 8 h	[33]
Streptomycin	60%	3/5	Audiometry 250–8000 Hz	Hearing threshold shift ≥ 20 dB in one frequency; ≥ 15 dB at two frequencies	10 weeks ± 6	Not defined	[41]
Streptomycin	19%	6/32	Audiometry 250–8000 Hz	Hearing threshold shift ≥ 20 dB in one frequency	11.5 weeks (range 2–107)	75 mg/kg/week	[29]
Tobramycin	30%	7/23	Electronystagmography with caloric irrigation	Reduced labyrinth excitability	Not defined	Not defined	[42]
Tobramycin	19%	19/102	Audiometry 250–8000 Hz	Hearing threshold shift ≥ 15 dB at two frequencies	8 days ± 3	1.7 mg/kg/8 h	[33]
Tobramycin	18%	5/28	Audiometry 250–20 kHz	ASHA 1994 definition	> 4 days	not defined	[31]

*Ref* reference, *IQR* interquartile range, *cVEMP* cervical vestibular-evoked myogenic potential

Aminoglycoside-induced mitochondrial damage is linked with the drugs’ antibiotic mechanism. Specifically, aminoglycosides bind to the A-site on the 16S ribosomal RNA of the 30S ribosome, inhibiting bacterial protein synthesis and killing the bacteria [7]. Shared ancestry between mitochondria and bacteria means that the mitochondrial ribosome is similar to the bacterial ribosome, and therefore susceptible to aminoglycoside binding. Aminoglycoside-induced disruption of mitochondrial protein synthesis is subsequently detected by innate cellular monitoring systems, initiating cell signalling cascades that result in HC death.

Cell death pathways are not well-defined in the inner ear. However, it is known that excessive mitochondrial ROS production and subsequent cellular oxidative stress is a key aspect of the process [8–10]. As a result, research aiming to mitigate ototoxicity has predominantly focused on antioxidant compounds, with equivocal and often conflicting outcomes [11]. These studies have not translated to changes in medical care, and in some cases, antioxidant use was associated with serious side effects, such as bleeding, pain, and gastric distress [11]. It is unclear why antioxidant therapies that have been effective in vitro have not achieved particularly good outcomes in vivo*.* However, normal ROS production is an integral part of cellular and physiological function; thus, antioxidant therapies can induce damaging reductive cell stress [12].

In addition to ROS production, the activation of c-Jun N-terminal kinases (JNK) and p38 mitogen-activated protein kinase (P38) has been reported in aminoglycoside-treated HCs; and various JNK and P38 inhibitors attenuate aminoglycoside-induced HC death [13–17]. However, JNK and P38 have critical homeostatic functions, meaning that inhibition can have toxic effects in other tissues, such as the liver and spiral ganglion neurons [18, 19]. Therefore, upstream molecules that drive the apoptosis-specific roles of JNK and P38 might represent better targets for ameliorating aminoglycoside-induced HC death. Recently, Tao et al. demonstrated that apoptosis signal-regulating kinase 1 (ASK1) was the only mitogen-activated protein kinase kinase kinase (MAP3K) to have significantly increased RNA expression after aminoglycoside treatment in HCs [20]. ASK1 is activated by redox imbalance and activates downstream MAP kinases, particularly JNK and P38. In addition, multiple ASK1 inhibitors have been developed, with encouraging results in human clinical trials [21]. Therefore, ASK1 inhibition may be a novel strategy for preventing aminoglycoside-induced HC death.

*Ask1* is expressed throughout the murine cochlear epithelium, including IHCs and OHCs [22–24] (data may be viewed at https://umgear.org/index.html?layout_id=f64f9c22&gene_symbol=map3k5&gene_symbol_exact_match=0). However, the role of ASK1 as a mediator of aminoglycoside-induced HC death has not been investigated. Furthermore, the role of ASK1 in normal auditory function has not been defined. Therefore, this study aimed to evaluate the importance of ASK1 in the mouse HC by characterising the auditory phenotype of *Ask1*^−/−^ mice and examining whether *Ask1*^−/−^ HCs are resistant to aminoglycoside toxicity. The utility of pharmacologically-mediated ASK1 inhibition was also tested for preventing aminoglycoside-induced HC death.

## Methods

Detailed methodology is provided in Supplementary Material 1.

### Study approval

The Murdoch Children’s Research Institute and Sunnybrook Research Institute Animal Ethics Committees approved procedures in project numbers A875, A904, and 21515. The Royal Children's Hospital (RCH) Human Research Ethics Committee approved sputum collection in project number 25054.

### Mice

*Ask1*^−/−^ mice were generated previously [43] and backcrossed for > 12 generations onto the C57BL/6 background. Wild type (WT) C57BL/6 mice were purchased from the Walter and Eliza Hall Institute of Medical Research (Parkville, Australia) and used as experimental controls. CD-1 mice were purchased from Charles River Laboratories (Raleigh, USA).

### Acoustic startle response (ASR) and auditory brainstem response (ABR)

ASRs were measured using the SR-LAB system (San Diego Instruments) as described in [44]. ABRs were measured using an evoked potentials workstation and BioSigRP Stimulate/Record System v4.4.1 (Tucker Davis Technologies). Sound pulses were presented to anaesthetised mice, in short bursts (100 μs duration, repeated 512 times), through a free-field magnetic speaker (model FF1, Tucker Davis Technologies) 10 cm from the mouse’s left ear. Hearing thresholds were defined as the lowest sound pressure level capable of eliciting a visible ABR.

### Tissue collection and analysis

Adult mice were euthanised by anaesthetic overdose and perfused with 10% NBF. Dissected cochleae were decalcified in 10% EDTA (7 days at 4 °C), oriented in 1% agarose in cryomolds (Sakura Finetek, Torrance, CA, USA) and paraffin-embedded. A microtome cut 2-µm sections parallel to the modiolus. Sections were stained with hematoxylin and eosin (H&E).

### Neonatal mouse neurosensory epithelium dissection, culture, and processing

See Ogier et al. 2019 protocol [45] for stepwise method. Pups were euthanised by decapitation and cochlear explants were collected for organotypic culture. For *Ask1*^−/−^-WT comparisons, explants were cultured overnight before fresh media was added containing neomycin or vehicle (DMSO/saline). For ASK1 inhibition experiments, explants were cultured 3–4 h, before being treated for 16 h with GS-444217 (supplied by Gilead Sciences, San Francisco, CA). Fresh media was added containing GS-444217 and the associated treatment. Explants were fixed and stained (as per Supplementary Material Table 1). For HC quantification, images from the explant base to apex were photo-stitched together and de-identified. Fiji software was used to draw two boxes (0.18 mm × 0.09 mm), which were aligned and overlaid either side of the explant mid-point. HCs were counted using the Fiji cell count tool, including cells with > half the cell body inside the rectangle boundary. The average HC number of the two boxes was recorded for each explant.

### Western blot analysis

Protein was extracted from three pooled explants per treatment, producing ~ 1 mg/ml protein per sample. Ten micrograms protein was denatured and separated in a precast Gel (Bio-Rad Cat#4,561,093). Protein was transferred onto a 0.45 µm pore PVDF membrane (Immobilon-P, Cat#IPVH00010). Antibodies were incubated as per Supplementary Material Table 1. Relative quantification of steady state protein levels was performed by first normalising to the loading control, and then to a non-treated negative control within each blot.

### Antibiotic minimum inhibitory concentration assay

Antibiotic efficacy was tested against a blood isolate *Pseudomonas aeruginosa* reference strain (American type culture collection, 27853-provided by the RCH Department of Microbiology) and two clinical isolates (0307 and 0315 provided by RCH Respiratory and Sleep Medicine, having been collected by the Australian Respiratory Early Surveillance Team for Cystic Fibrosis and isolated by RCH pathology). A 96-well plate containing serial 1:2 dilutions of amikacin, tobramycin or neomycin and GS-444217 was inoculated with *P. aeruginosa* and incubated at 37 °C for 16 h. The minimum inhibitory concentration (MIC) was defined as the amount of antibiotic required to prevent bacterial metabolism of resazurin, as measured by fluorescence (540 nm excitation and 580 nm emission) on an Infinite M200 Pro plate reader (Tecan life Sciences).

### Statistics

ABR and ASR data was compared between groups for each frequency/volume tested using unpaired *T* tests (with the Holm Sidak correction for multiple comparisons). Protein levels were compared using a standard two-way ANOVA with Sidak’s multiple comparison test. A standard two-way ANOVA was used to identify changes in MICs and post hoc *T* tests were performed using the two-stage step-up false discovery method of Benjamini, Krieger, and Yekutieli. These analyses were performed using GraphPad prism (version 7.0a for Mac). HC counts were analysed with a three-way analysis of variance, before pairwise Fisher individual tests of the differences of means were performed to ascertain *p* values for strain/treatment. This analysis was performed in Minitab software (version 17 for Windows) under the guidance of Dr. Sue Finch, Melbourne University Statistical Consulting Platform.

## Results

### Ask1^−/−^ cochlear morphology and HCs appear normal

**Fig. 1:**
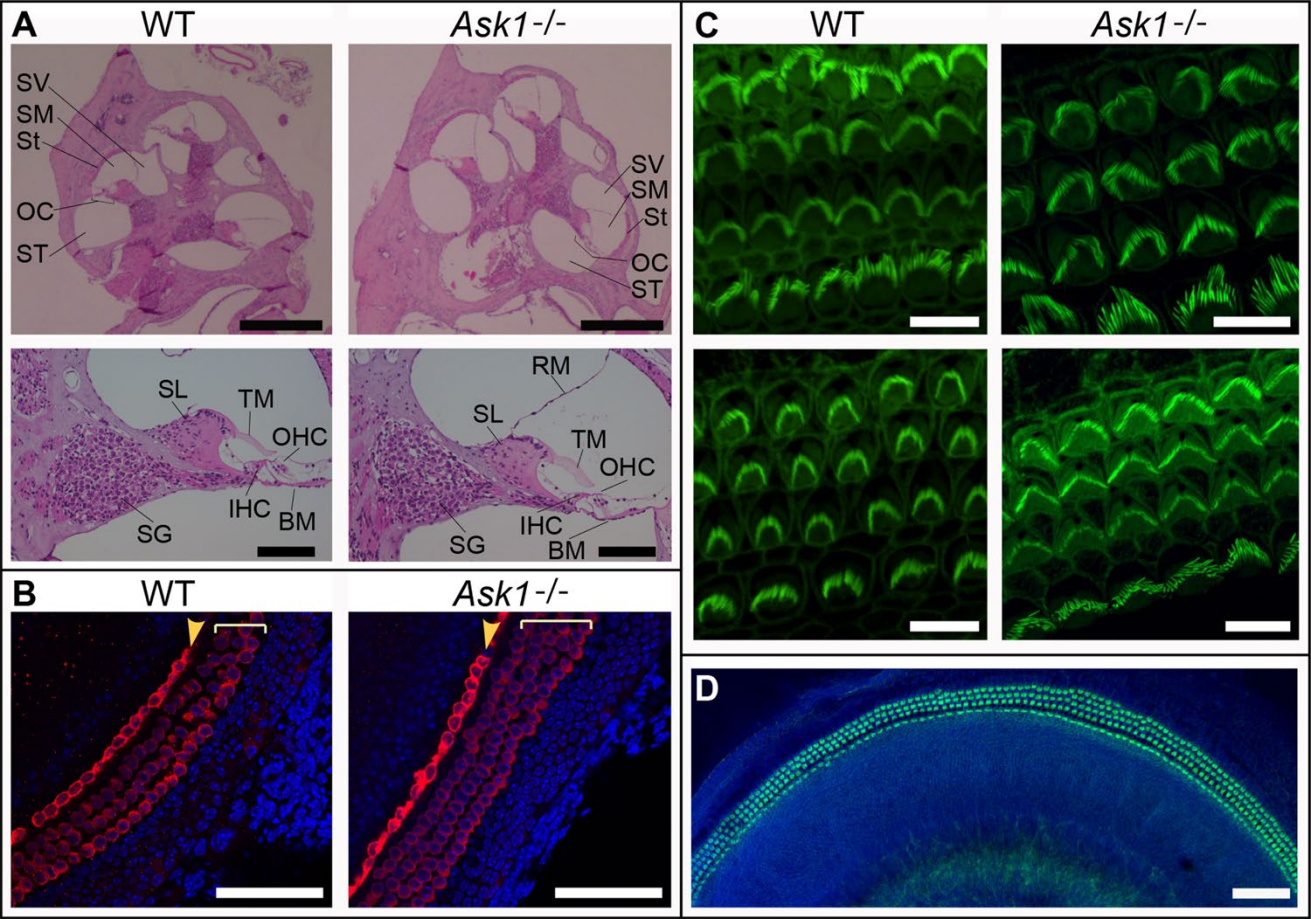
Analysis of cochlear morphology and hair cell patterning in *Ask1*^−/−^ mice. **A** Representative H and E stained cochlear mid-modiolar sections from 8-week-old wild type (WT) and *Ask1*^−/−^ mice (upper panels). Scale bar represents 500 μm. Lower panels show higher magnification images of a single mid-turn in the mid-modiolar sections. Scale bar represents 100 μm. SV: scala vestibuli, SM: scala media, St: stria vascularis, OC: organ of Corti, ST: scala tympani. SL: spiral limbus, TM: tectorial membrane OHC: outer hair cells, SG: spiral ganglion (neurons,) IHC: inner hair cell, BM: basilar membrane, RM: Reissner’s membrane. *n* = 3 mice per genotype. **B** Representative confocal images of WT and *Ask1*^−/−^ neonatal (P4) cochlear explants that were cultured overnight. Red = Myosin VIIa stain used to identify hair cells and Blue = nuclear DAPI counterstain. The single row of inner hair cells (yellow arrowhead), and three rows of outer hair cells (square brackets) are clearly delineated. Scale bars = 70 μm. **C** Phalloidin staining of P3 explants (green) shows stereocilia bundles with graded changes in length and width, from apex (upper panels) to base (lower panels). Scale bar = 10 μm. **D** Phalloidin staining of the upper-mid region of an *Ask1*^−/−^ explant, counterstained with DAPI (blue) also demonstrates hair cell patterning in the *Ask1*^−/−^ explant. Scale bar = 70 μm

No gross deformities were noted during dissection, nor in sectioned *Ask1*^−/−^ cochleae, compared to WT controls (Fig. 1A). Myosin VIIa-staining of cochlear explants dissected from neonatal (P4) mice showed HCs in typical formation, in both WT and *Ask1*^−/−^ explants (Fig. 1B). Phalloidin staining of P3 explants showed stereocilia on the apical HC surfaces of both WT and *Ask1*^−/−^ explants (Fig. 1C).

### Functional assessment of hearing in Ask1^−/−^ mice

**Fig. 2:**
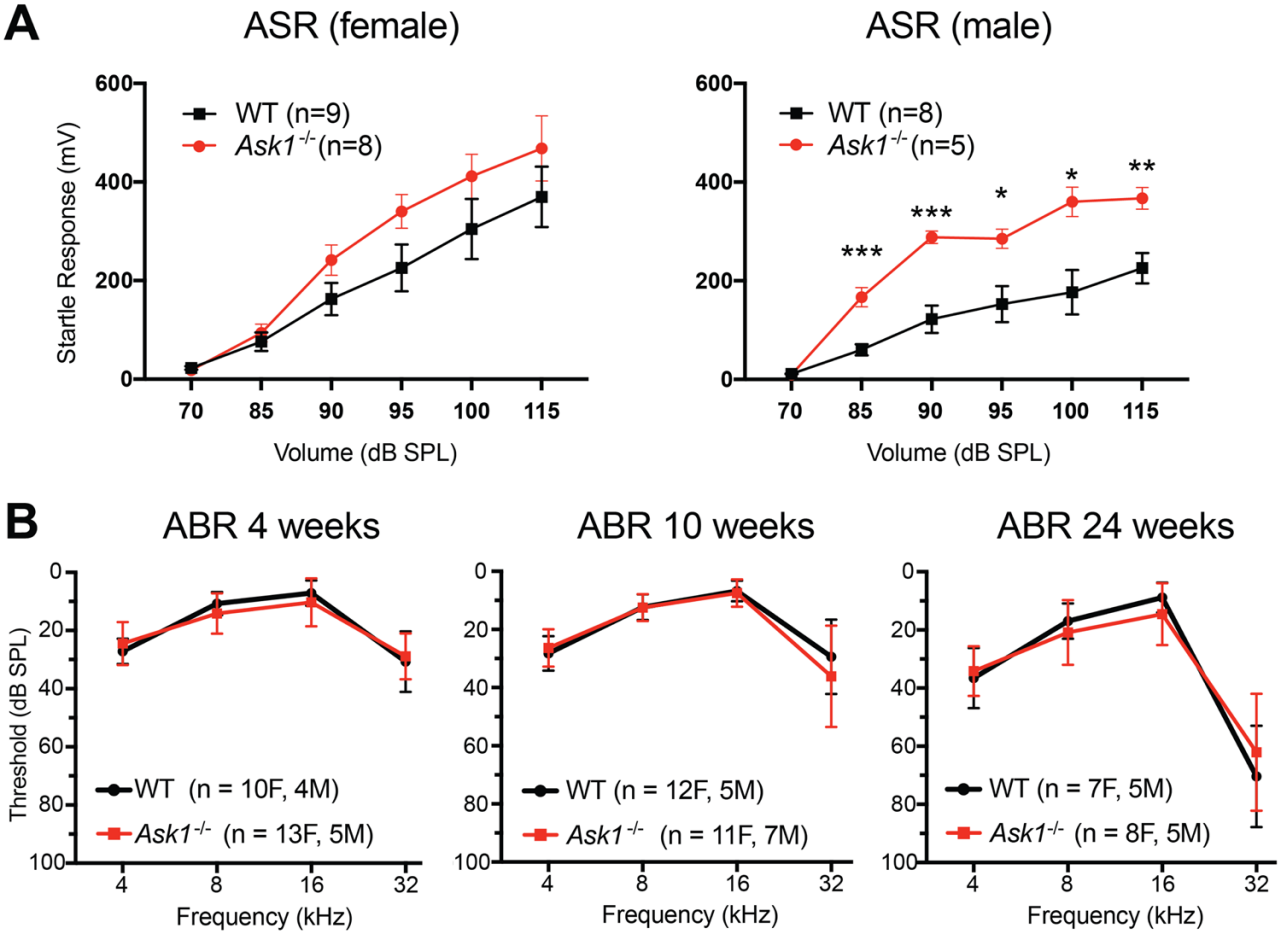
The *Ask1*^−/−^ acoustic startle response and auditory brainstem response. **A** *Ask1*^−/−^ mice (red) tended to have a stronger acoustic startle response than wild type (WT) controls (black). This difference was statistically significant in the male cohort, as detected using multiple unpaired *T* tests, * indicates *p* < 0.05, ** indicates *p* < 0.01, *** indicates *p* < 0.001. Error bars represent SEM. **B** The auditory brainstem response thresholds of *Ask1*^−/−^ (red) and WT (black) mice. There was no significant difference between the average hearing thresholds of *Ask1*^−/−^ and WT mice at 4, 10, and 24 weeks of age. Age-related, high-frequency hearing loss was evident in both *Ask1*^−/−^ and WT mice at 24 weeks of age. F = female, M = male, *n* = sample size. Error bars = S.D

The acoustic startle response (ASR) of male *Ask1*^−/−^ mice was significantly higher than that of WT controls, suggesting that male *Ask1*^−/−^ mice are acoustically hypersensitive (Fig. 2A). A similar trend was present in the data from female *Ask1*^−/−^ mice, although this did not reach statistical significance. As factors unrelated to hearing, such as muscle strength, emotional status, or memory function can contribute to the ASR [46, 47], the auditory brainstem response (ABR) was performed to assess hearing in the *Ask1*^−/−^ strain. ABR testing showed that there was no difference between *Ask1*^−/−^ and WT hearing thresholds when measured between 4 and 32 kHz at 4, 10, and 24 weeks of age (Fig. 2B).

### Ask1^−/−^ HCs are resistant to neomycin-induced cell death

**Fig. 3:**
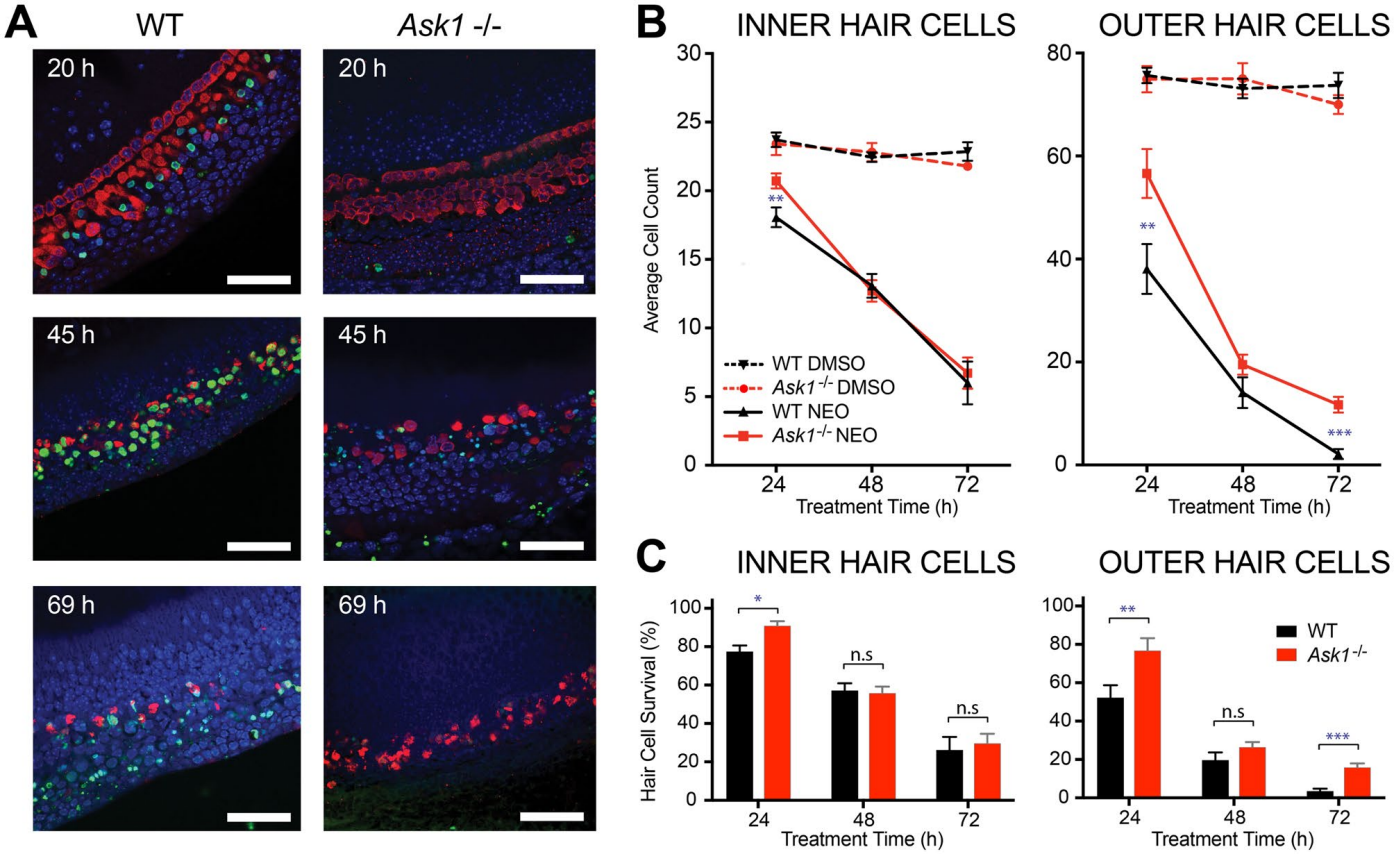
Effect of *Ask1* knockout on neomycin-induced hair cell death. **A** Representative images of wild type (WT) P3 cochlear explants (left) compared with *Ask1*^−/−^ cochlear explants (right) treated for 20, 45, or 69 h with 1 mM of neomycin. Green = TUNEL stain (dying cells), blue = DAPI (nuclei), red = Myosin VIIa (hair cells). Images were collected from the mid-turn of each explant. Scale bar = 50 μm. **B** Average number of Myosin VIIa-labelled inner and outer hair cells after 24, 48, or 72 h treatment with either vehicle (DMSO) or 1 mM neomycin. The *n* values for 24, 48, or 72 h respectively are: WT DMSO *n* = 7, 9, 7; *Ask1*^−/−^ DMSO *n* = 6, 8, 7; WT neomycin *n* = 8, 7, 6 and *Ask1*^−/−^ neomycin *n* = 7, 5, 7. Mean ± SEM shown (* *p* < 0.05, ** *p* < 0.01 per fisher individual means test). **C** Hair cell counts presented as percentage survival based on average hair cell counts in the DMSO control group. Mean ± SEM shown (* *p* < 0.05, ** *p* < 0.01, *** *p* < 0.001, n.s = not significant as per a standard unpaired *T* test)

Cochlear explants were cultured overnight before the addition of 1 mM neomycin or vehicle (DMSO). Notable OHC death (as indicated by positive TUNEL staining) occurred 20 h after treatment commenced (Fig. 3A). After 45 h, there was a notable loss of Myosin VIIa-positive HCs and TUNEL staining was prevalent. By 69 h, neomycin caused near-complete loss of Myosin VIIa positive HCs. In comparison, *Ask1*^−/−^ explants showed a markedly different response to neomycin treatment. There was no observable HC TUNEL stain at the 20 h time point, very little at the 45 h time point and none at the 69 h time point (Fig. 3A). Furthermore, the nuclear compartment of remaining HCs appeared less fragmented at the 20- and 45 h time points in the *Ask1*^−/−^ group when compared to time-matched controls. However, at the 69 h time point, the nuclei of Myosin VIIa-positive HCs were significantly condensed or fragmented in the *Ask1*^*−/−*^ explants (Fig. 3A). These qualitative observations suggested that HC death is delayed in neomycin-treated *Ask1*^*−/−*^ explants. This observation also indicated that TUNEL staining was not the most suitable means to assess hair cell death, because hair cells that were lost were not always TUNEL-positive at the time points captured. In contrast, Myosin VIIa immunohistochemistry provided robust and specific hair cell labelling. As a result, the subsequent methodology for quantifying hair cell survival was based on Myosin-VIIa staining.

To quantify HC survival, we determined the number of Myosin VIIa positive cells in two 180 μm × 90 μm fields of view in the cochlear mid-turn. Over a time-course of 3 days, there was no significant difference between the HC counts in WT and *Ask1*^−/−^ explants that had been treated with vehicle only (*p* = 0.4 (IHC), 0.7 (OHC), two-way ANOVA) (Fig. 3B). In comparison, significant genotype dependant differences were observed after neomycin treatment. At the 24 h time point, 77% ± 3% of IHCs remained in neomycin-treated WT explants, which was significantly less than the neomycin-treated *Ask1*^−/−^ explants (91% ± 2%) (mean ± SEM, *p* = 0.006) (Fig. 3B, C). In contrast, there was no significant difference between WT and *Ask1*^−/−^ IHC counts at the 48- and 72 h time points (*p* = 0.8 and 0.7 respectively) (Fig. 3B, C). Neomycin-induced OHC death was more pronounced than IHC death in both WT and *Ask1*^−/−^ explants. However, *Ask1*^−/−^ OHCs showed resistance to neomycin toxicity, as evidenced by a significantly slower rate of loss when compared to neomycin-treated WT OHCs (Fig. 3B). After 24 h of neomycin treatment, only 52% ± 7% of WT OHCs remained compared with 77% ± 6% of *Ask1*^−/−^ OHCs (*p* = 0.02). The difference was not significant at the 48 h time point, with 19% ± 4% of WT OHCs persisting compared to 26% ± 3% *Ask1*^−/−^ OHCs (*p* = 0.2). After 3 days of neomycin treatment, only 3% ± 1% of the OHCs remained in WT explants compared to 16% ± 2% in the *Ask1*^−/−^ explants (*p* = 0.0002) (Fig. 3C).

### ASK1 inhibition protects WT HCs from neomycin toxicity

Having demonstrated that a genetic-mediated reduction in ASK1 affords protection against neomycin-induced HC death in vitro, we hypothesised that a small-molecule inhibitor of ASK1 (GS-444217) would also protect WT (C57BL/6) HCs against neomycin toxicity in vitro.

**Fig. 4:**
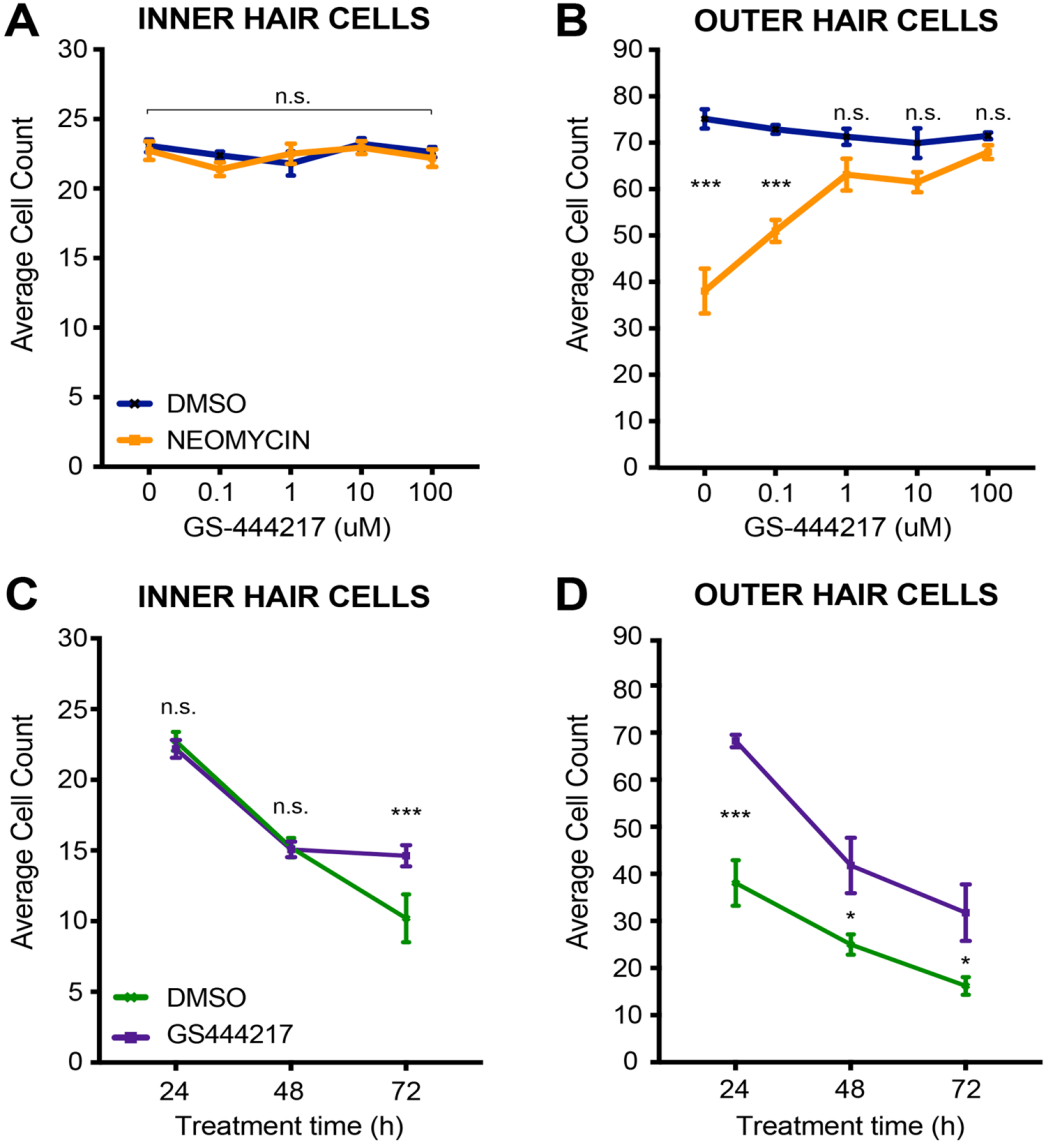
Effect of ASK1 inhibitor GS-444217 on hair cell survival in neomycin-treated cochlear explants. Quantitation of Myosin VIIa-positive inner (**A**) and outer (**B**) hair cells from explants pre-treated with GS-444217 (0–100 μM) for 16 h before 24 h incubation with vehicle (DMSO) or 1 mM neomycin. Counts for each explant represent the average of two 180 μm × 90 μm fields of view, taken from the explant mid-turn. *N* values for 0, 0.1, 1, 10, and 100 μM GS-444217-treated explants respectively = 7, 9, 5, 7, 9 (DMSO) and 8, 6, 7, 9, 8 (neomycin). Mean with SEM shown (*** *p* < 0.001, n.s = not statistically significant as per one way ANOVA). **C** and **D** The average number of Myosin VIIa-positive inner (**C**) and outer (**D**) hair cells for explants that had been pre-treated (16 h) with vehicle (DMSO) (green) or 100 μM GS-444217 (purple) before the addition of 1 mM neomycin for a 3-day time-course analysis. *N* values for 24-, 48-, and 72 h time points respectively = 8, 5, 5 (DMSO) and 9, 7, 6 (GS-444217). Mean with SEM shown (*p < 0.05, *** *p* < 0.001, n.s = not statistically significant)

To test if treatment with GS-444217 could compromise HC survival, cochlear explants were cultured for 24 h with GS-444217 doses ranging from 0 to 100 μM. There was no evidence of increased HC death as a result of GS-444217 treatment (one-way ANOVA; OHC counts *p* = 0.36, IHC counts *p* = 0.27) (Fig. 4A, B, blue line).

Next, we investigated if treatment with GS-444217 could protect against aminoglycoside-induced HC death in vitro. Cochlear explants were pre-treated for 16 h with GS-444217 (0 μM-100 μM) or vehicle (DMSO), and then incubated for a further 24 h with the addition of 1 mM neomycin (Fig. 4A, B, orange line). As observed previously (Fig. 3), IHCs were highly resistant to neomycin treatment, with less than 1% of IHCs lost after 24 h neomycin treatment (Fig. 4A). Overall, there was no significant difference (*p* = 0.69) in the number of IHCs in neomycin-treated explants that had been pre-treated with either vehicle (22.7 ± 0.7, *n* = 7) or GS-444217 (23.1 ± 0.5, *n* = 9) at the 24 h time point. In contrast, GS-444217 pre-treatment markedly improved OHC survival in neomycin-treated explants, with concentrations over 1 μM GS-444217 significantly attenuating OHC death (Fig. 4B, orange line). For explants pre-treated with vehicle (DMSO) before being exposed to 1 mM neomycin, only 38.1 ± 4.8 Myosin VIIa-positive OHCs remained at the 24 h time point. When this number is normalised to untreated controls, a survival rate of 51% is indicated. OHC number increased to 51 ± 2.5 (68% survival) with a pre-treatment of 0.1 μM GS-444217, and 1 μM GS-444217 treatment resulted in 63 ± 2.5 OHCs surviving (92% survival). At a concentration of 100 μM GS-444217 pre-treatment, 68 ± 1.4 OHCs survived, representing 95% cell survival when compared to the DMSO treated controls (Fig. 4B).

Over a 3-day neomycin treatment period, 100 μM GS-444217 afforded significant protection for both IHCs and OHCs (Fig. 4C, D). There was no difference in IHC survival at the 24- and 48 h time points. However, after 72 h of neomycin treatment, an average of 10.2 ± 1.7 IHCs were counted in the field of view for vehicle pre-treated explants, compared to 15.3 ± 0.6 in the GS-444217 pre-treated explants (*p* < 0.001). The effect of GS-444217 pre-treatment was more pronounced in the OHCs, protecting against neomycin treatment at every time point tested (Fig. 4D). After 24 h, the average OHC counts for vehicle pre-treated explants was 38.1 ± 4.8 compared to 68.2 ± 1.3 in the GS-444217 pre-treated explants (*p* < 0.001). At 48 h, only 25 ± 2.2 OHCs persisted in the vehicle pre-treated explants compared to 41.8 ± 5.9 in the GS-444217 pre-treated group (*p* = 0.04) and after 72 h neomycin treatment, 16.2 ± 1.9 OHCs remained in the vehicle group, compared to 32.1 ± 4.2 in the GS-444217 group (*p* = 0.01).

### Protective effect of GS-444217 in cochlear explants

**Fig. 5:**
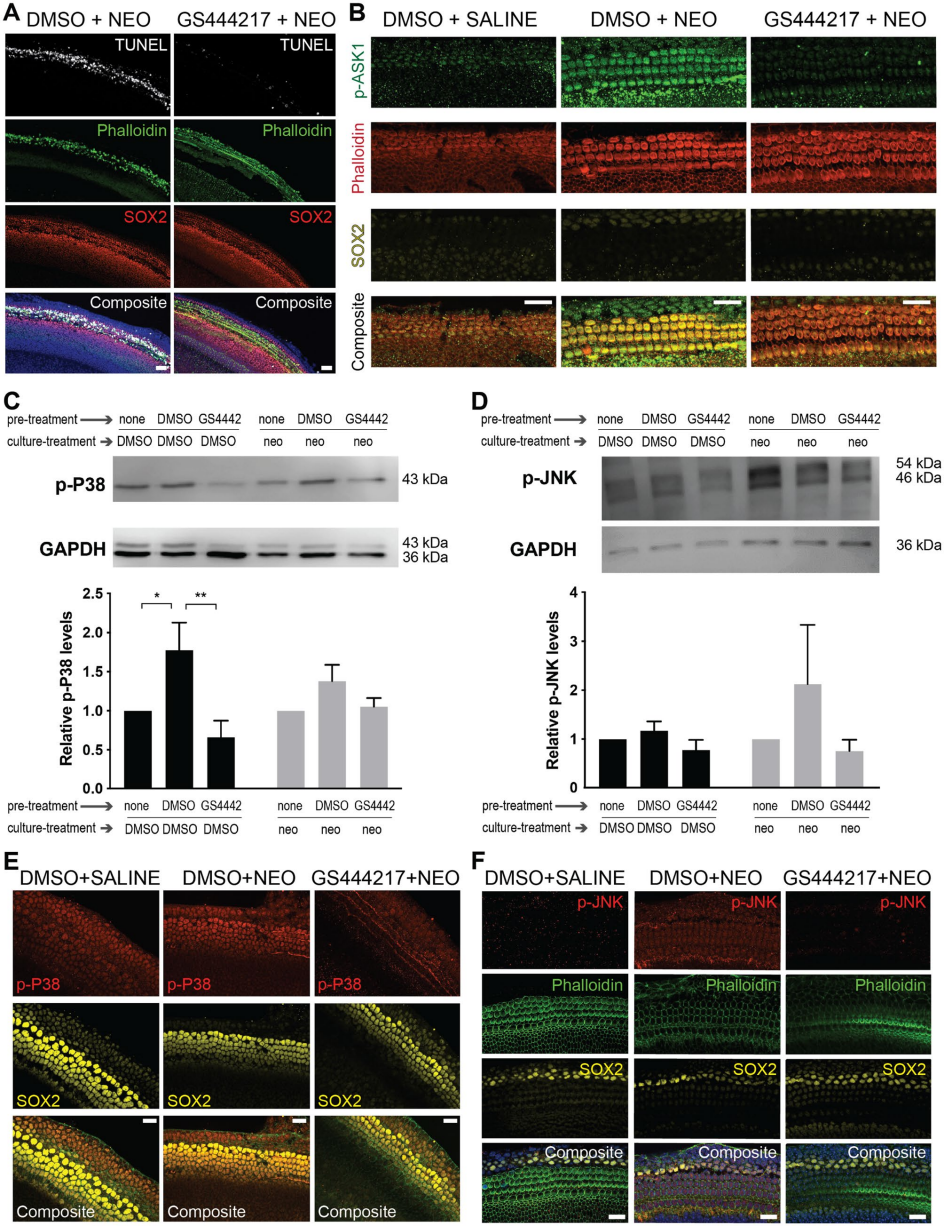
Molecular effects of GS-444217 treatment in cochlear explants. **A** TUNEL stained, wild type (WT) CD-1 cochlear explants (P3) that had been pre-treated with DMSO (left) or 10 μM GS-444217 (right), and subsequently treated with 1 mM neomycin for 24 h. White = TUNEL (dying cells), green = phalloidin (highlighting hair cells), red = SOX2 (support cells), blue = DAPI (nuclei). Scale bar = 40 μm. **B** P3 WT explants that had been pre-treated with DMSO or 10 μM GS-444217 before receiving 1 mM neomycin treatment (or saline control) labelled with p-ASK1 (green), phalloidin (red), and SOX2 (yellow). Scale bar = 20 μm. **C** Representative western blot stained for p-P38 and loading control (GAPDH) followed by protein quantification (*n* = 3). Note: the faint band above GAPDH is p-P38. **D** Representative western blot stained for p-JNK1, 2 and 3, and loading control (GAPDH); followed by protein quantification (*n* = 3). JNK 1, 2 and 3 are present in two isoforms (p54 and p46). Each sample contains protein extracted from three pooled explants that had been pre-treated with either DMSO or 100 μM GS-444217 and subsequently co-treated with either DMSO or 1 mM neomycin (neo) for 24 h. Relative protein levels in DMSO and neomycin-treated samples were calculated by first normalising to the loading control and then to the correlated non-treated control (pre-treatment → none). Error bars = SEM, * = *p* < 0.05, ** = *p* < 0.01, as detected using a standard two-way ANOVA. **E** Support cells in cochlear explants treated with 1 mM neomycin for 12 h are positive for p-P38; however, this signal appears reduced in explants pretreated with 10 μM GS-444217. **F** Cochlear explants treated with 1 mM neomycin for 4 h and labelled with a p-JNK antibody indicate that 10 μM GS-444217 pre-treatment attenuates pro-apoptosis JNK phosphorylation in cochlear hair cells. For E and F, red = p-P38/p-JNK, green = phalloidin (hair cells), yellow = SOX2 (support cells), blue = DAPI, and scale bar = 20 μm

To investigate how GS-444217 reduces neomycin-induced hair cell death, we performed immunohistochemistry and western blot analysis. As previously observed in our *ASK1* genetic knockout experiments (Fig. 3), ASK1 inhibition using GS-444217 limited canonical apoptosis (indicated by TUNEL staining) (Fig. 5A). Immunohistochemistry also showed that GS-444217 pre-treatment limited neomycin-induced ASK1 phosphorylation in cochlear hair cells (Fig. 5B). Western blot analysis of WT cochlear explants that were pre-treated with GS-444217 and then cultured for 24 h with either vehicle (DMSO) or 1 mM neomycin indicated a significant reduction of p-P38 when explants were pre-treated with GS-444217 in DMSO co-treated culture conditions (Fig. 5C). A similar trend of reduced p-P38 in GS-444217 pre-treated explants was observed in explants cultured with neomycin. Likewise, the p-JNK signal tended to be lower in explants that had been pre-treated with GS-444217, for both DMSO and neomycin-treated explants; however, this was not statistically significant. (Fig. 5D). We then performed a time course experiment to elucidate the impact of GS-444217 pre-treatment on JNK and P38 phosphorylation in cochlear explants, from 1 to 24 h post neomycin treatment (Supplementary Figs. 1–3). P38 phosphorylation was most notable in the supporting and epithelial cells of the cochlear explant (Fig. 5E), whereas the strongest p-JNK signal was observed in hair cells 4 h post-neomycin treatment (Fig. 5F). A faint p-JNK signal was also detected in the supporting cells of neomycin-treated cochlear explants (Supplementary Fig. 3). Notably, a strong p-JNK signal was present in the axons of spiral ganglia in all treatment groups, correlating with previous observations that p-JNK has a homeostatic role for axonal development and maintenance (Supplementary Fig. 3) [19, 48, 49].

### GS-444217 does not impair antibiotic efficacy against Pseudomonas aeruginosa isolates

**Fig. 6:**
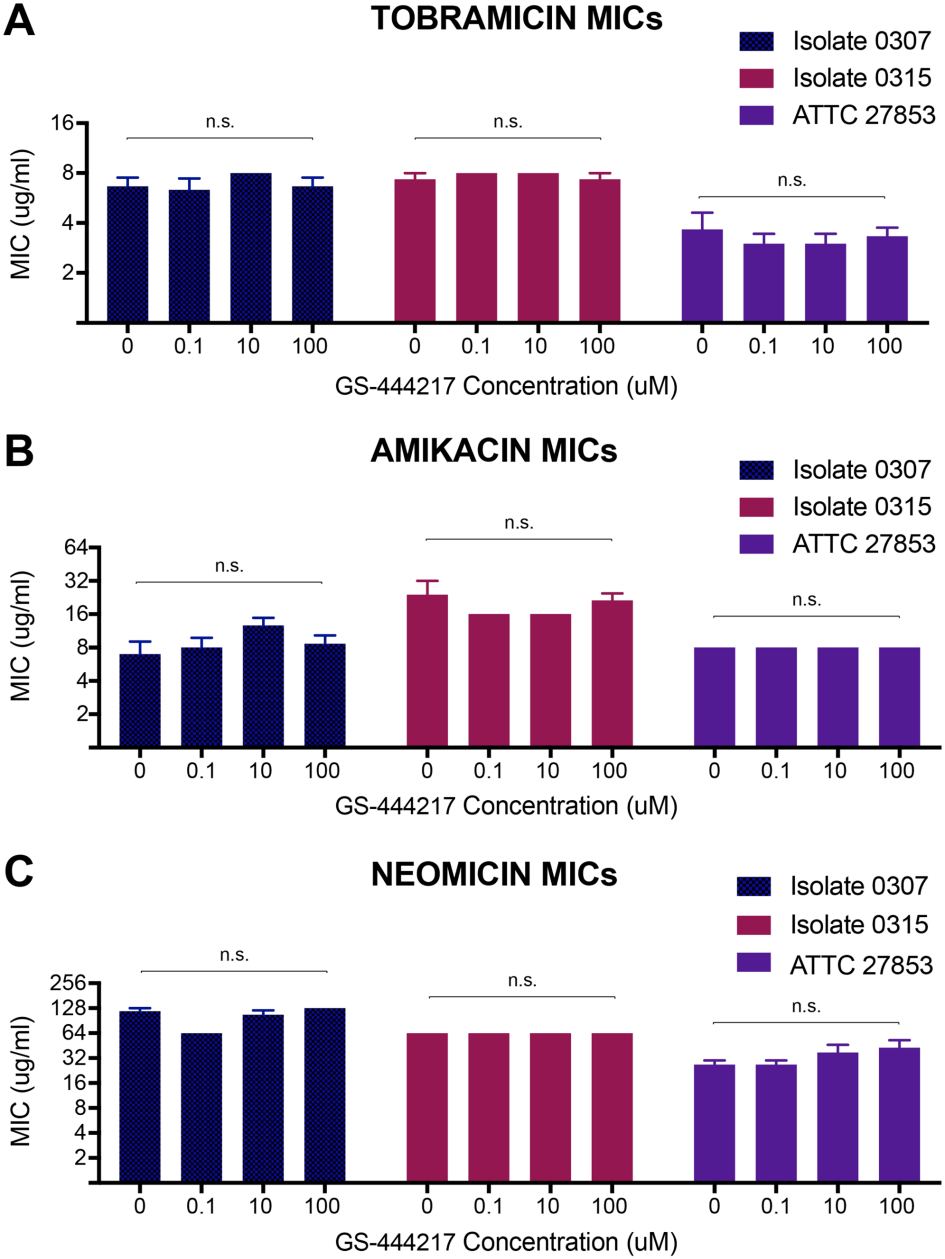
The effect of GS-444217 on the minimum inhibitory concentration required of tobramycin, amikacin, or neomycin to prevent *Pseudomonas aeruginosa* metabolism of resazurin. MIC’s were calculated using the broth dilution test for **A** tobramycin, **B** amikacin and **C** neomycin. *n* = three broth dilution plates per isolate for each antibiotic, with each plate containing two replicates for each GS-444217 concentration. Error bars = SEM. Note, no error bars indicate that calculated MIC was the same for all three plates

To evaluate the possibility that GS-444217 could have an off-target effect upon antibiotic action, the minimum inhibitory concentration (MIC) of amikacin, tobramycin, and neomycin against *Pseudomonas aeruginosa* (*P. aeruginosa*) was determined. *P. aeruginosa* is the most common cause of chronic lung infections in individuals with cystic fibrosis and these individuals are particularly susceptible to aminoglycoside-induced hearing loss, due to long-term aminoglycoside treatment [50]. One reference strain (ATTC 27853) and two clinical isolates (0307 and 0315) were utilised. Tobramycin and amikacin were tested because they are the most clinically utilised aminoglycosides for *P. aeruginosa* treatment. Neomycin was also tested as an aminoglycoside frequently used in the laboratory. The broth dilution test demonstrated that GS-444217 co-treatment did not significantly change the minimum antibiotic concentration required for any antibiotic tested to inhibit resazurin metabolism in all three *P. aeruginosa* strains (Fig. 6).

## Discussion

This study utilised *Ask1*^*−/−*^ mice and a small-molecule inhibitor of ASK1 (GS-444217) to test if the downregulation of ASK1 attenuates aminoglycoside-induced HC death. Both genetic and pharmacological approaches demonstrated that ASK1 has an important role in the process of HC death and indicated that ASK1 inhibition could limit aminoglycoside ototoxicity.

Prior to this work, the effect of *Ask1* knockout on auditory function had not been investigated. We used a combination of histology, immunohistochemistry, and functional ABR testing to demonstrate that *Ask1* knockout does not compromise the peripheral auditory structures or hearing thresholds of mice. However, male *Ask1*^*−/−*^ mice presented with a hypersensitive startle response when compared to WT controls, and a similar trend was observed in the female cohort. It is difficult to predict what the underlying cause of the unusually strong ASR might be, because auditory signal integration and efferent control is not well understood [51]. High ASR’s generally indicate neural processing dysfunction in an auditory or psychiatric disorder [52], whilst factors such as muscle strength, emotional status, and memory function can also affect the ASR [46, 47]. Notably, abnormal behaviour in *Ask1*^*−/−*^ mice has been previously reported, including hyperactivity, enhanced motor coordination, and significantly increased dopaminergic transmission [53]. ASK1 deficiency has also been implicated with impaired sensory gating in individuals with schizophrenia [54] and an ASK1 polymorphism is associated with reduced cognitive empathy in men [55]. Combined, these observations suggest that ASK1 may play a role in neuronal signalling. Whilst this has not yet been directly investigated, A*sk1*^*−/−*^ mice could prove useful for further exploration of neuronal development and signal processing mechanisms.

The major aim of this study was to investigate the potential role of ASK1 in aminoglycoside-induced HC death. Analysis of *Ask1*^*−/−*^ cochlear explants demonstrated that *Ask1* deficiency significantly attenuates neomycin-induced OHC death. *Ask1* deficiency also afforded some protection against neomycin-induced IHC death; however, this was less pronounced. Subsequent experiments using GS-444217 produced results that correlated with the genetic ablation data. However, HC survival appeared slightly higher in the pharmacological data. The main methodological difference between these independent experiments was the addition of DMSO, used as a vehicle for GS444217. DMSO is a potent antioxidant that has previously been shown to increase hair cell survival [56–58]. Therefore, the DMSO used in the GS444217 pre-treatment/control may account for any improvement of HC survival during pharmacological experiments. Overall, GS-444217 pre-treatment resulted in significantly more OHCs surviving 3 days of neomycin treatment, when compared with DMSO pre-treated controls. However, neither genetic knockout nor pharmacological inhibition of ASK1 completely attenuated HC death. This suggests that alternate mechanisms, such as MAPK independent caspase activation, may be removing HCs. Alternatively, damaged HCs can be phagocytosed by cochlear-resident macrophages or supporting cells [59, 60]. Western blot analysis indicated that P38 and JNK phosphorylation occurred in GS-444217-treated explants, which correlates with previous studies that utilised *Ask1*^−/−^ mice and ASK1 inhibitors in alternate disease models [43, 61, 62]. Therefore, it is likely that ASK1 independent mechanisms are able to activate the P38 and JNK pathways in cochlear explants. Nevertheless, the increased HC survival observed in GS-444217-neomycin-treated explants was significant, especially given the non-physiological conditions in which cochlear explants are cultured. In particular, the experimental dose of 1 mM neomycin—applied directly to the HCs in vitro*—*is unlikely to represent the concentration of aminoglycoside found in vivo in cochlear endolymph. This experimental dose is used in vitro to achieve clear results in a limited experimental timeframe, because optimal HC survival is less than one week in vitro [45]. However, a dose achieving cochlear levels of 1 mM neomycin would not be considered safe for use in humans. Likewise, other clinically utilised aminoglycosides do not reach such high concentrations in vivo. For example, a study of 113 individuals receiving amikacin over a 2-week period demonstrated that average peak plasma concentrations did not exceed 17 μM amikacin [63]. Moreover, the stria vascularis acts as an important blood-barrier in vivo, limiting the amount of aminoglycoside entering the endolymph. Kinetic studies in rats have shown that a gentamycin plasma level of 13–14 μM achieves only 3.1 μM in the perilymph and 2.5 μM in the endolymph [64]. Similar results have been observed in the guinea pig, with intravenous gentamycin (300 mg/kg) achieving a peak plasma concentration of 1.1 mM, but only 0.3 mM in the cochlear lymph 3 h after treatment [65]. Therefore, the otoprotective effect of GS-444217 may be stronger in vivo, where HCs are likely to be exposed to significantly lower aminoglycoside concentrations than those used in this in vitro study*.*

To further investigate the utility of pharmacological inhibition of ASK1 as a protective strategy against aminoglycoside ototoxicity, we tested whether GS-444217 altered the efficacy of aminoglycosides against *P. aeruginosa* in vitro. Notably, these data indicated that 100 μM GS-444217 did not impair antibiotic efficacy, whereas 1 μM GS-444217 significantly attenuated neomycin-induced HC death*.* In combination, these results suggest that GS-444217 could be used to prevent aminoglycoside-induced HC death without impacting primary aminoglycoside care. Whilst this must be assessed in vivo, the promising safety of ASK1 inhibitors taken orally in clinical trials, combined with the observation that GS-444217 does not affect aminoglycoside antibiotic efficacy in vitro, implies that ASK1 inhibition could be applied as an otoprotective strategy in the clinic. Further studies are now needed to confirm that GS-444217 and other promising ASK1 inhibitors do not impair antibiotic efficacy against common infections, such as Group *B streptococcus*, *E*. *coli*, *Staphylococcus aureus*, or Enterobacteriaceae infection. These organisms often cause sepsis in neonates and subsequent aminoglycoside treatment is the greatest risk factor for these newborns developing hearing loss [66–68].

Collectively, our data provide compelling evidence that ASK1 inhibition may be a valid strategy for mitigating aminoglycoside-induced hearing loss in humans. Whilst previous studies have demonstrated the safety of ASK1 inhibition in human clinical trials [21], strong in vivo efficacy data is required to provide the evidence base for clinical testing of ASK1 inhibition in aminoglycoside-treated individuals. In particular, the inhibitor dosing schedule required to achieve meaningful auditory protection must be determined. Based on the safety results of human clinical trials to date, we anticipate the minimum dose of an ASK1 inhibitor will have no effect on the innate immune response; however, this needs to be fully evaluated in vivo*.* In this study, we have demonstrated that 1 μM of GS-444217 significantly attenuates HC death caused by 1 mM neomycin treatment. However, a trend of improved HC survival was also achieved using only 0.1 μM GS-444217. It is difficult to predict the exact GS-444217 dosing regimen that will be required to achieve similar cochlear concentrations in vivo; however, previous studies have achieved GS-444217 plasma concentrations markedly higher than 1 μM. For example, a single oral dose of 50 mg/kg GS-444217 resulted in plasma levels of 92.8 mM in mice [69]. In rats, a single oral dose of 30 mg/kg, achieved peak plasma concentrations approaching 100 μM, although GS-444217 plasma levels declined to approximately 0.1 μM after 24 h [70]. This indicates that twice daily GS-444217 dosing may be beneficial. Other ASK1 inhibitors have also been tested in vivo, with 30 mg/kg of GS-459679 achieving plasma concentrations of approximately 6 μM in mice, 6.5 h after administration [71]. Unfortunately, a conspicuous limitation for in vivo testing of ASK1 inhibition as an otoprotective strategy is that mice appear to be intrinsically resistant to aminoglycoside-induced HC death [72]. Therefore, additional models are required to generate the necessary pre-clinical data. The pig may be appropriate, as the porcine metabolic rate, immune system, cochlea, and auditory range closely mimic that of humans [73, 74]. In addition, swine models of cystic fibrosis exist that may be valuable for studying the progression of infection and the efficacy of aminoglycoside antibiotics when an adjuvant ASK1 inhibitor is used [75]. A porcine model could also provide information regarding the benefits of ASK1 inhibition in regard to other aminoglycoside toxicities, such as aminoglycoside nephron and neurotoxicity.

This work provides a strong foundation to justify the development of pre-clinical models that may lead to the development of an adjuvant otoprotective therapy. Such a therapy would have far-reaching implications, as aminoglycoside ototoxicity is a significant health issue, not only for those directly affected, but also clinical health care providers and the economy in general. Avoiding the distressing and lifelong co-morbidities of acquired hearing loss, such as loneliness, impaired mobility, mental fatigue, cognitive decline, and increased mortality risk, is critically important for the health of an individual. From an economic perspective, mitigating aminoglycoside ototoxicity will reduce the financial burden of acquired hearing loss within the national health, education, and welfare systems. In the case of critical healthcare, the prevention of aminoglycoside ototoxicity may also improve the utility of aminoglycoside antibiotics. At present, clinicians must consider the risk of permanent hearing loss against the risk to life when prescribing an aminoglycoside treatment. However, reducing ototoxic outcomes would ease the burden experienced by clinicians during this decision-making process and potentially allow for the wider application of aminoglycoside therapy.

## Supplementary Information

Below is the link to the electronic supplementary material.

Supplementary file1 (DOCX 8807 KB)

Supplementary file2 (DOCX 30 KB)

## Data Availability

Important data generated or analysed during this study is included in this published article. Further information is available from the corresponding author on reasonable request.
