# Correction to: ASK1 is a novel molecular target for preventing aminoglycoside‑induced hair cell death

**DOI:** 10.1007/s00109-022-02203-5

**Published:** 2022-05-10

**Authors:** Jacqueline M. Ogier, Yujing Gao, Eileen M. Dunne, Michael A. Wilson, Sarath C. Ranganathan, Gregory H. Tesch, David J. Nikolic Paterson, Alain Dabdoub, Rachel A. Burt, Bryony A. Nayagam, Paul J. Lockhart

**Affiliations:** 1 Bruce Lefroy Centre, Murdoch Children’s Research Institute, Parkville, VIC Australia; 2 Department of Paediatrics, University of Melbourne, Parkville, VIC Australia; 3 Sunnybrook Research Institute, Toronto, ON Canada; 4 Pneumococcal Research Group, Murdoch Children’s Research Institute, Parkville, VIC Australia; 5 Infection and Immunity, Murdoch Children’s Research Institute, Parkville, VIC Australia; 6 Department of Respiratory Medicine, The Royal Children’s Hospital, Parkville, VIC Australia; 7 Department of Nephrology, Monash Health and Monash University Centre for Inflammatory Diseases, Clayton, VIC Australia; 8 Department of Anatomy and Neuroscience, The University of Melbourne, Parkville, Australia; 9 Department of Audiology and Speech Pathology, The University of Melbourne, Parkville, VIC Australia

## Correction to: Environmental Science and Pollution Research https://doi.org/10.1007/s00109-022-02188-1

The numbers 432 and 433 found in the 6th paragraph of the Discussion section is not included in this paper and should be removed.

The Original article has been corrected.

