# Supplementary material for: ASK1 is a novel molecular target for preventing aminoglycoside-induced hair cell death
Source: J Mol Med (Berl). 2022 Apr 26;100(5):797–813. doi: 10.1007/s00109-022-02188-1 (PMC9110505; doi:10.1007/s00109-022-02188-1)

**Supplementary Figure 1. p-JNK staining in neonatal mouse cochlear hair cells.**

P3 CD-1 mouse cochlear explants were pre-treated with 10 μM GS-444217 or DMSO for 16 hours before being treated with 1 mM neomycin or saline control (1-24 hours). SOX2 staining of the support cells (yellow) and phalloidin staining of hair cell stereocilia (green) was used to provide landmarks for selecting three z stack slices. Red = p-JNK, blue = DAPI. Scale bar = 20 μm.


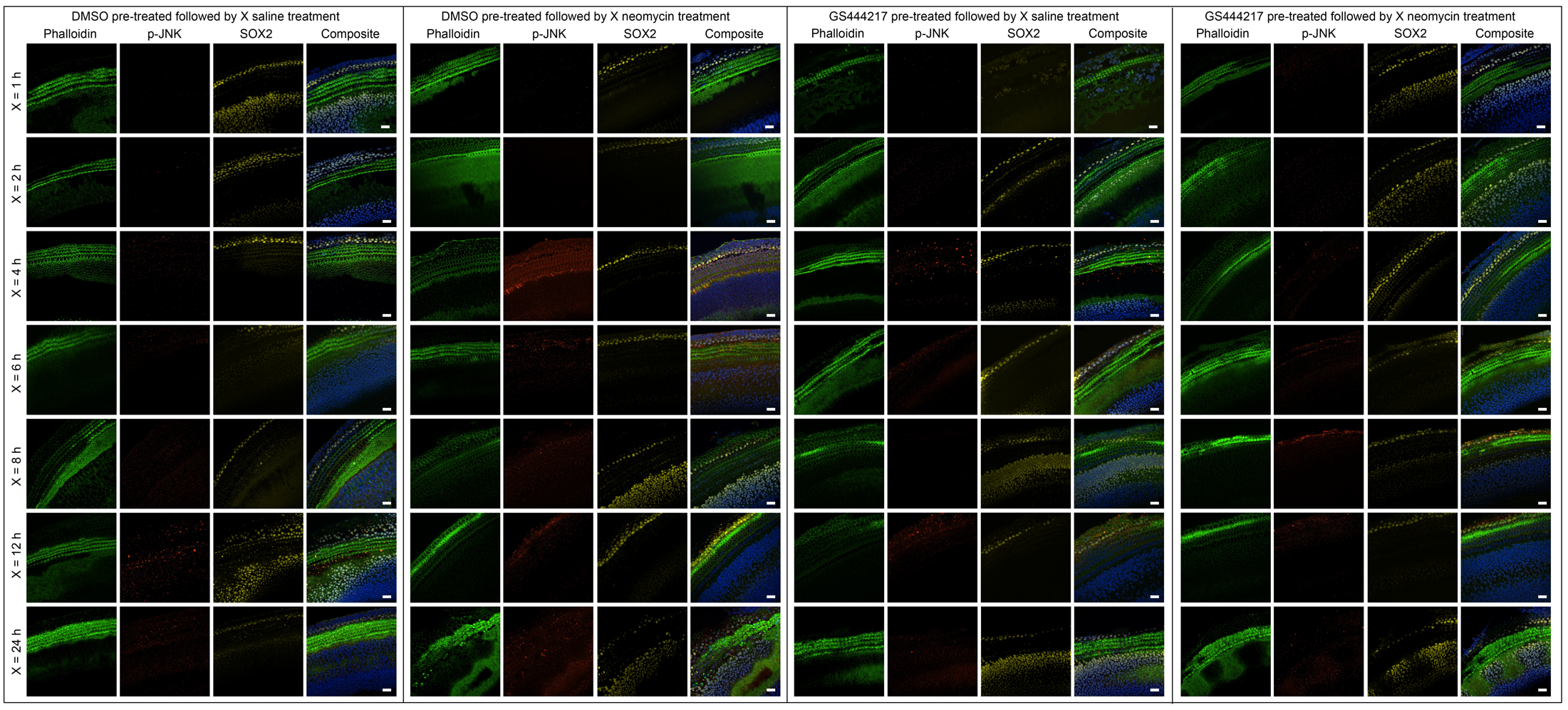


**Supplementary Figure 2. p-P38 staining in neonatal mouse cochlear hair cells.**

P3 CD-1 mouse cochlear explants were pre-treated with 10 μM GS-444217 or DMSO for 16 hours before being treated with 1 mM neomycin or saline control (1-24 hours). SOX2 staining of the support cells (yellow) and phalloidin staining of hair cell stereocilia (green) was used to provide landmarks for selecting three z stack slices. Red = p-P38, blue = DAPI. Scale bar = 20 μm.


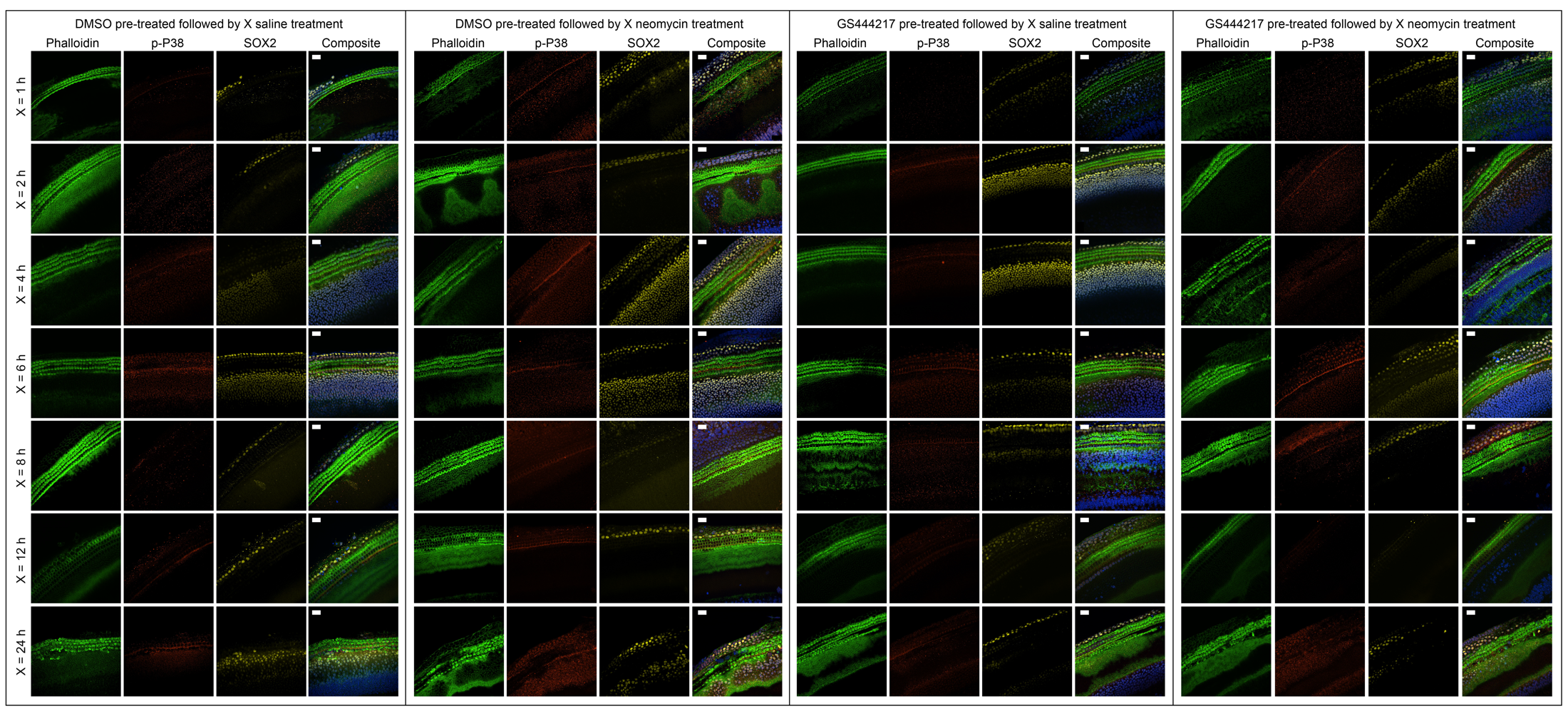


**Supplementary Figure 3. p-JNK staining in the supporting structures of the neonatal mouse cochlea.**

P3 CD-1 mouse cochlear explants were pre-treated with 10 µM GS-444217 or DMSO for 16 hours before being treated with 1 mM neomycin or saline control for 4 hours. Z stack images contain six slices, collected from the base of the explant to the apical region of support cells (identified by SOX2 staining). Red = P-JNK, green = SOX2 and blue = DAPI. Scale bar = 20 μm.


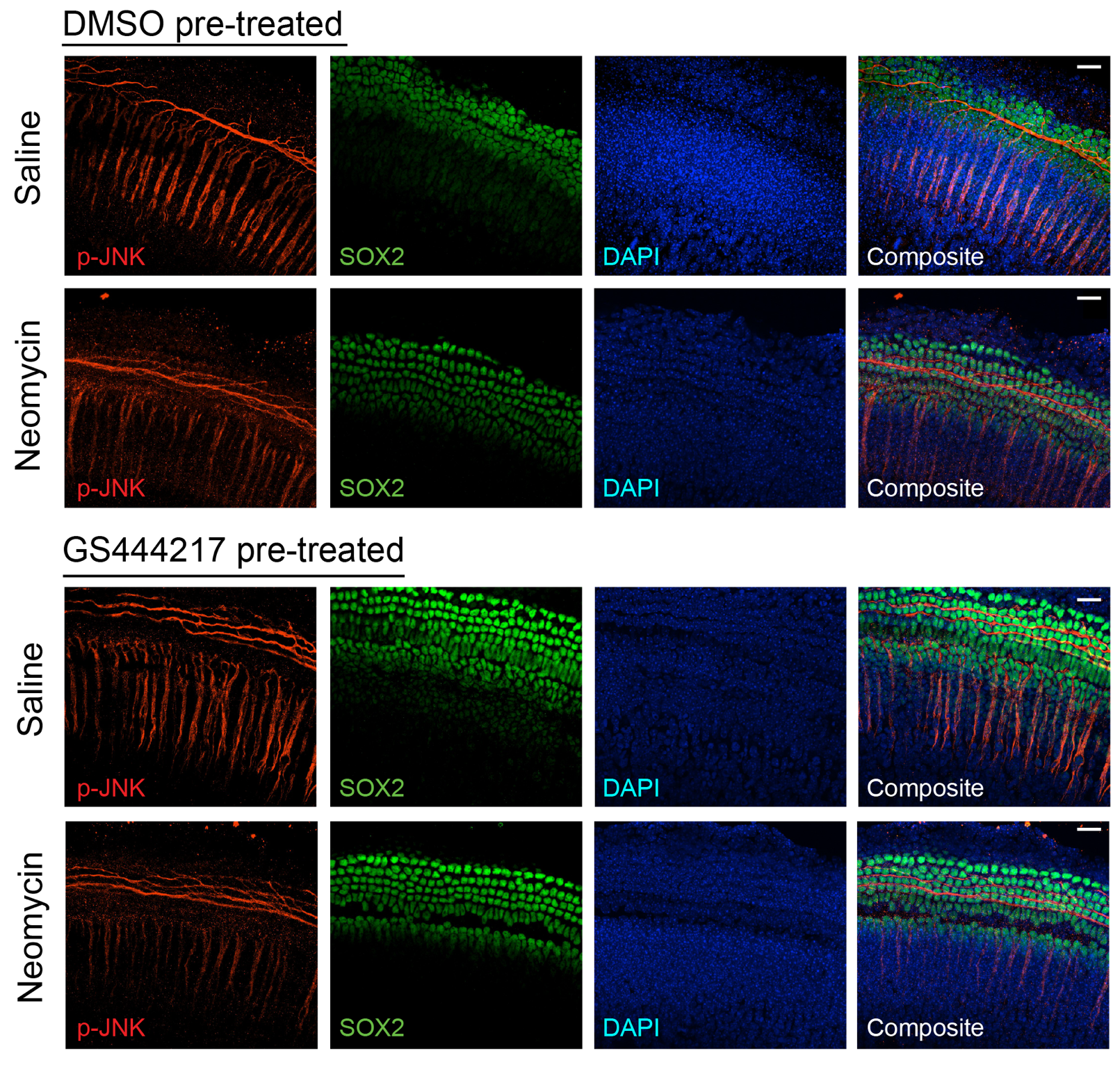

Supplement: Supplementary file 1 — Supplementary file1 (DOCX 8807 KB) [file 109_2022_2188_MOESM1_ESM.docx]
