# Supplementary material for: ASK1 is a novel molecular target for preventing aminoglycoside-induced hair cell death
Source: J Mol Med (Berl). 2022 Apr 26;100(5):797–813. doi: 10.1007/s00109-022-02188-1 (PMC9110505; doi:10.1007/s00109-022-02188-1)
Supplement: Supplementary file 2 — Supplementary file2 (DOCX 30 KB) [file 109_2022_2188_MOESM2_ESM.docx]

**Supplementary Material: Detailed experimental procedures**

*Ethics approval*

The Murdoch Children’s Research Institute (MCRI) Animal Ethics Committee approved mouse procedures in project numbers A875 and A904, in compliance with the Australian Code of Practice for the Care and Use of Animals for Scientific Purposes, 2013. The Sunnybrook Research Institute (SRI) Animal Care Committee approved mouse procedures in protocol number 21515, conforming with Canadian Council on Animal Care requirements. The Royal Children's Hospital (RCH) Human Research Ethics Committee approved the collection of sputum specimens in project number 25054. Clinical specimens were collected in accordance with the National Health and Medical Research Council’s Statement on Ethical Conduct in Human Research, 2007 (2018 Update) and the Australian Code for the Responsible Conduct of Research, 2018.

*Mice*

*Ask1*^-/-^ mice were generated as previously described (1) and backcrossed for more than 12 generations onto the C57BL/6 background. Wild type (WT) C57BL/6 mice were purchased from the Walter and Eliza Hall Institute of Medical Research (Parkville, Australia) and used as experimental controls. C57BL/6 and *Ask1*^-/-^ mice were housed at MCRI in individually ventilated micro-isolator cages, with up to five mice per enclosure (Tecniplast, VA, Italy). The ambient temperature was 23 °C and relative humidity was 30-60%. The automated light cycle was 12 hours on, 12 hours off. Mice had free access to Barastoc mouse chow (Ridley AgriProducts, VIC, Australia) and autoclaved water. Crinkled paper, chew sticks, and sunflower seeds provided environmental enrichment. Gravid CD-1 mice were purchased from Charles River Laboratories (North Carolina, USA) and briefly housed at SRI in individually ventilated micro-isolator cages (Allentown, NJ, USA). The ambient temperature was 18-22 °C and relative humidity was 30-60%. The automated light cycle was 12 hours on, 12 hours off. Mice had free access to Teclad Global 18% Protein diet 2918 (Envigo, IND, USA) and reverse osmosis chlorinated water. Crinkled paper and a cotton nestlet provided environmental enrichment.

*Genotyping*

Genomic DNA was extracted from mouse ear clips as previously described (2). Standard PCR and agarose electrophoresis were used to identify the wild type (WT) allele (563 bp, primers cttgatctgctggtccacgtcc and gacactaaacccaccctgcctcc) or *Ask1* knockout allele (312 bp, cttgatctgctggtccacgtcc and caacgggttcttctgttagtcc).

*Acoustic Startle Response (ASR)*

ASRs were measured using the SR-LAB system (San Diego Instruments) as previously described (3). Mice were restrained in a Perspex capsule in a sound attenuating box. After a one-minute habituation period with background white noise (70 dB SPL), pulses of varying sound level were delivered using a pseudo-randomised program. Six pulses of white noise were delivered at background levels (70 dB SPL), 85, 90, 95, and 100 dB SPL, and 16 pulses at 115 dB SPL. The time between each pulse was 3 to 8 seconds. A piezoelectric accelerometer created a voltage output based on the mouse’s response to each sound, which was recorded using SR‐Lab software (San Diego Instruments). The highest and lowest response for each sound level was deleted before the average was taken from all ASRs.

*Auditory Brainstem Response (ABR)*

Mice were anesthetised with 80 mg/kg ketamine and 20 mg/kg xylazine via intraperitoneal injection (3). Sedation was sufficient when the mouse no longer exhibited the Pedal withdrawal reflex. An additional 40 mg/kg ketamine and 10 mg/kg xylazine was administered 10 minutes after the initial dose if the mouse retained a pedal reflex. Refresh night-time eye ointment was applied to protect the mouse’s eyes and a heat cabinet (Tecniplast) maintained the mouse’s temperature at 37 °C. In a custom Coulbourn habitest isolation cubicle containing a faraday cage, sub-dermal stainless-steel electrode needles (4mm, S06666-0, Rochester Electro-Medical) were inserted at the vertex of the mouse’s skull (positive), left cheek (negative), and left hind leg (ground). Computer generated pulses of sound were produced by an evoked potentials workstation (Tucker Davis Technologies) in conjunction with the BioSigRP Stimulate/Record System v4.4.1. Clicks (white noise of mixed frequency spectrum 0-50 kHz) or pure tones (4 kHz, 8 kHz, 16 kHz and 32 kHz) were presented in short bursts (100 μs in duration, repeated 512 times), through a free-field magnetic speaker (model FF1, Tucker Davis Technologies) 10 cm from the mouse’s left ear. Hearing thresholds were defined as the lowest sound pressure level capable of eliciting a visible ABR (calculated as the average of all 512 repeats). The maximum intensity of sound tested was 100 dB SPL, decremented in 5 dB SPL steps.

*Tissue Collection and Analysis*

Adult mice were euthanised by anaesthetic overdose (400 mg/kg ketamine and 80 mg/kg xylazine in saline, i.p). After respiratory arrest, the mouse’s pedal response was checked before the thoracic cavity was carefully opened. A 21-gauge needle was inserted into the left ventricle and the right atrium was perforated. Each animal was perfused for five minutes with PBS, followed by five-minutes of 10% neutral buffered formalin (NBF). Cochleae were collected and stored in 10% NBF. For sectioning, cochleae were washed in Tris-buffered saline and decalcified in 10% EDTA (on a roller for 7 days at 4 °C). Cochleae were oriented in 1% agarose (in PBS) in 10 mm × 10 mm × 5 mm cryomolds (Sakura Finetek, Torrance, CA, USA) before being paraffin-embedded. A microtome was used to cut 2 µm sections parallel to the modiolus. Sections were stained with hematoxylin and eosin (H&E) and imaged using a Leica DM 1000 stereo microscope, DFC450 camera, and Leica Application Suite v 3.8 software.

*Neonatal mouse neurosensory epithelium dissection, culture and processing*

These methods are fully described in a stepwise manner in Ogier et al. 2019 (4). In brief, mouse pups (P3-4) were euthanised by decapitation. Under sterile conditions, the temporal bones were dissected and the vestibular apparatus, otic capsule, cochlear wall, and stria vascularis were removed before the neurosensory epithelium was unwound from the cochlear modiolus. Cochlear explants were cultured on organotypic membranes (Merck Millipore Milli Cell) at 37 °C, 5% CO_2_ in neurobasal-A medium (containing N2 Supplement, L-glutamine and D-glucose, Life Technologies). For *Ask1*^-/-^ and wild type mouse comparisons, explants were cultured overnight before fresh media was added containing neomycin or vehicle (DMSO/saline). For ASK1 inhibition experiments, explants were cultured for 3-4 hours, before being treated for 16 hours with GS-444217 or vehicle (DMSO/saline) (kindly supplied by Gilead Sciences, San Francisco, CA and subsequently purchased from cedarlane labs Ontario, Canada, A20517-1). Fresh media was added containing GS-444217 and the associated treatment. Explants were fixed in 4% PFA, washed in PBS and blocked in 0.1% Triton X-100 in PBS containing 2% normal goat serum. The primary and secondary antibodies were applied in 20 μl of blocking solution, as per Supplementary Material Table 1. When the *in situ* cell detection kit was used, explants were washed in blocking solution before 7ul of the cell death detection solution was added and incubated at 37° C for 1 hour. Explants were then washed and briefly incubated with phalloidin. ProLong Gold antifade (Life Technologies) was used to mount slides, which were air-dried and sealed with nail varnish. Confocal imaging was performed using a Laser Scanning Microscope 780 (Zeiss) in conjunction with Zen Black digital imaging software (Zeiss).

Supplementary Material Table 1. Antibodies, stains and kits used for immunofluorescence and Western blot analysis. IF = antibody used for immunofluorescence, WB = antibody used for Western blot analysis.

| Antibody/Target | Catalogue Details | Dilution (& use) | Incubation |
| --- | --- | --- | --- |
| Goat Anti-Rabbit IgG (conjugated with Alexa Fluor 488 or 594) | Sapphire Bioscience.  AB150085, AB150080 | 1/500 (IF) | 2 h at room temperature or overnight at 4 °C |
| *In situ* cell death detection kit (TUNEL) | Sigma Aldrich. 11684795910 | 1:9 Enzyme to label solution (IF) | 1 h at 37 °C |
| Myosin-VIIa | Sapphire Bioscience. PTS-25-6790-C050 | 1/500 (IF) | 1 h at room temperature or overnight at 4 °C |
| Phalloidin (conjugated with  Alexa Fluor 488 or 594) | Invitrogen.  A12379, A12381 | 1/180 (IF) | 8-10 min at room temperature |
| Phospho-JNK 1, 2 & 3 (conjugated with Alexa Fluor 488) | Abcam. AB201862 | 1/1000 (WB) | Overnight at 4 °C |
| Phospho-JNK 1, 2 & 3 | Invitrogen. PA5104906 | 1/200 (IF) | Overnight at 4 °C |
| Phospho-P38 | Cell Signalling #9211 | 1/1000 (WB) | Overnight at 4 °C |
| Phospho-P38 | Abcam. AB4822 | 1/200 (IF) | Overnight at 4 °C |
| GAPDH (control) | Cell Signalling #5174 | 1/10 000 (WB) | Overnight at 4 °C |
| β-actin (control) | Sigma-Aldrich A5441 | 1/10 000 (WB) | Overnight at 4 °C |
| Goat anti-rabbit (HRP conjugate) | Jackson Immuno #111035003 | 1/20 000 (WB) | 1 h at room temperature |
| SOX2 | Santa Cruz Technology. SC-365964 | 1/200 | Overnight at 4 °C |

*Hair Cell Quantification*

Methods for hair cell quantification are also described in a stepwise manner in Ogier et al. 2019 (4). An Olympus IX70 fluorescent microscope with Evolution VF cooled monochrome 12-bit camera captured images from the explant base to apex (imaging software Qcapture pro V 6.0). Adobe Photoshop CC.2015 was used to photo-stitch images together. Images were de-identified and Fiji software was used to draw two boxes (0.18mm x 0.09mm). The boxes were aligned to the explant and overlaid onto the image, either side of the explant mid-point. Hair cells within each box were manually counted using the Fiji cell count tool, including partial cells with more than half the cell body inside the rectangle boundary. The average hair cell number of the two boxes was then recorded for each explant.

*Western Blot Analysis*

Three explants from each treatment were pooled in protein extraction buffer (10 mM Tris-HCL, pH 7.5, 2% SDS and 1x Protease inhibitor (Sigma-Aldrich, Cat# S8820) on wet ice. A 1 ml syringe with 23-guage needle was used to begin homogenisation before lysates were sonicated using a probe sonicator (Branson). Pooled explants produced ~ 1 mg/ml protein sample, as estimated using the bicinchoninic acid assay (BCA) (Pierce, Cat# 23225) and a Bovine Serum Albumin (BSA) standard curve). Ten micrograms of total protein was denatured at 95° C for five minutes in 1x Novex™ Tris-Glycine SDS Sample Buffer (Thermo Scientific, Cat#LC2676) and 1% β-mercaptoethanol (Sigma-Aldrich, Cat#M6250) and then separated in a 4-20% gradient Mini-PROTEAN® TGX™ Precast Gel (Bio-Rad Cat#4561093), with 1x Novex™ Tris-Glycine SDS Running Buffer (Thermo Scientific, Cat#LC2675). Protein was transferred onto a 0.45 µm pore PVDF membrane (Immobilon-P, Cat#IPVH00010) using an overnight 10 V wet transfer (transfer buffer: 20% ethanol and 1x Novex™ Tris-Glycine SDS Running Buffer). Membranes were blocked in 5% skim milk/ TBST (10 mM Tris-HCl (pH 7.5), 150 mM NaCl and 0.05% Tween-20) for two hours at room temperature and then washed in TBST. Primary and secondary antibodies were diluted in TBST and incubated on the membrane as per Supplementary Material Table 1. Protein distribution was visualised using the Image Quant LAS4000 imager (GE Healthcare) and the Enhanced Chemiluminiscence Substrates kit (Bio-Rad, Cat#170-5061). Staining and imaging of the blot was repeated for control antibodies (Table 2). Relative quantification of steady state protein levels was performed by first normalising to the loading control, and then to a non-treated negative control within each blot.

*Antibiotic minimum inhibitory concentration assay*

Antibiotic efficacy was tested against three *Pseudomonas aeruginosa* isolates. A blood isolate reference strain (American type culture collection, 27853) was provided by the RCH Department of Microbiology. Two clinical isolates (0307 and 0315) were provided by the RCH department of Respiratory and Sleep Medicine. Isolates 0307 and 0315 were collected by the Australian Respiratory Early Surveillance Team for Cystic Fibrosis (AREST CF: <http://www.arestcf.org>) during routine hospital visits and *P. aeruginosa* was isolated by RCH pathology.

A sterile loop was used to streak *P. aeruginosa* from frozen stocks onto Luria Bertani agar plates which were incubated overnight at 37 °C, 5% CO_2_. A colony from each plate was used to inoculate 10 mL Luria-Bertani broth. The inoculated broth was incubated overnight and then diluted 1:100 in fresh broth. The diluted culture was incubated until an optical density of 0.1 at 600nm (approximately 582 cells per ml) was achieved. 30 μl of this inoculum was added to each well of a 96 well plate containing serial 1:2 dilutions of amikacin, tobramycin or neomycin and GS-444217. Antibiotic concentrations ranged from 0-512 μg/ml and GS-444217 concentrations were 0-100 μM, diluted in broth containing 700 μM of resazurin (Sapphire bioscience). Plates were incubated at 37 °C for 16 hours. The minimum inhibitory concentration was defined as the amount of antibiotic required to prevent bacterial metabolism of resazurin, as measured by fluorescence (540 nm excitation and 580 nm emission) on an Infinite M200 Pro plate reader (Tecan life Sciences).

*Statistical analysis*

ABR and ASR data was compared between groups for each frequency/volume tested using unpaired T-tests (with the Holm Sidak correction for multiple comparisons). Protein levels were compared using a paired, one-way Wilcoxon test. A standard two-way ANOVA was used to identify changes in MICs and post-hoc t-tests were performed using the two-stage step-up false discovery method of Benjamini, Krieger and Yekutieli. These analyses were performed using GraphPad prism software (version 7.0a for Mac). Hair cell counts were analysed with a three-way analysis of variance, before pairwise Fisher individual tests of the differences of means was performed to ascertain p-values for strain/treatment. This analysis was performed in MINITAB Software (version 17 for Windows) under the guidance of Dr Sue Finch, Melbourne University Statistical Consulting Platform.

**References:**

1. Ma FY, Tesch GH, Nikolic-Paterson DJ. ASK1/p38 signaling in renal tubular epithelial cells promotes renal fibrosis in the mouse obstructed kidney. Am J Physiol Renal Physiol. 2014 Dec;307(11):F1263–73.

2. Laird PW, Zijderveld A, Linders K, Rudnicki MA, Jaenisch R, Berns A. Simplified mammalian DNA isolation procedure. Nucleic Acids Res. 1991 Aug;19(15):4293.

3. Ogier JM, Carpinelli MR, Arhatari BD, Symons RCA, Kile BT, Burt RA. CHD7 deficiency in “Looper,” a new mouse model of CHARGE syndrome, results in ossicle malformation, otosclerosis and hearing impairment. PLoS One. 2014;9(5):e97559.

4. Ogier JM, Burt RA, Drury HR, Lim R, Nayagam BA. Organotypic Culture of Neonatal Murine Inner Ear Explants. Front Cell Neurosci. 2019 May 3;13:895.
